# A Malaria Diagnostic Tool Based on Computer Vision Screening and Visualization of *Plasmodium falciparum* Candidate Areas in Digitized Blood Smears

**DOI:** 10.1371/journal.pone.0104855

**Published:** 2014-08-21

**Authors:** Nina Linder, Riku Turkki, Margarita Walliander, Andreas Mårtensson, Vinod Diwan, Esa Rahtu, Matti Pietikäinen, Mikael Lundin, Johan Lundin

**Affiliations:** 1 Institute for Molecular Medicine Finland, University of Helsinki, Helsinki, Finland; 2 Department of Public Health Sciences, Karolinska Institute, Stockholm, Sweden; 3 Department of Computer Science and Engineering, University of Oulu, Oulu, Finland; Queensland University of Technology, Australia

## Abstract

**Introduction:**

Microscopy is the gold standard for diagnosis of malaria, however, manual evaluation of blood films is highly dependent on skilled personnel in a time-consuming, error-prone and repetitive process. In this study we propose a method using computer vision detection and visualization of only the diagnostically most relevant sample regions in digitized blood smears.

**Methods:**

Giemsa-stained thin blood films with *P. falciparum* ring-stage trophozoites (n = 27) and uninfected controls (n = 20) were digitally scanned with an oil immersion objective (0.1 µm/pixel) to capture approximately 50,000 erythrocytes per sample. Parasite candidate regions were identified based on color and object size, followed by extraction of image features (local binary patterns, local contrast and Scale-invariant feature transform descriptors) used as input to a support vector machine classifier. The classifier was trained on digital slides from ten patients and validated on six samples.

**Results:**

The diagnostic accuracy was tested on 31 samples (19 infected and 12 controls). From each digitized area of a blood smear, a panel with the 128 most probable parasite candidate regions was generated. Two expert microscopists were asked to visually inspect the panel on a tablet computer and to judge whether the patient was infected with *P. falciparum*. The method achieved a diagnostic sensitivity and specificity of 95% and 100% as well as 90% and 100% for the two readers respectively using the diagnostic tool. Parasitemia was separately calculated by the automated system and the correlation coefficient between manual and automated parasitemia counts was 0.97.

**Conclusion:**

We developed a decision support system for detecting malaria parasites using a computer vision algorithm combined with visualization of sample areas with the highest probability of malaria infection. The system provides a novel method for blood smear screening with a significantly reduced need for visual examination and has a potential to increase the throughput in malaria diagnostics.

## Introduction

Accurate malaria diagnosis is a key to successful management of the disease; yet in the African region, in 2012, up to 40% of suspected malaria cases attending public health facilities did not receive a diagnostic test [1]. In some regions, fewer than 10% of patients under the age of five presenting with fever are tested [2]. Effective diagnostics improves the management of patients with malaria, supports differential diagnosis regarding fever, and is essential for malaria surveillance reporting. Also, inappropriate use of costly first-line artemisinin-based combination therapies is reduced resulting in prevention of adverse effects, drug resistance and an improved cost-benefit ratio for the management of febrile symptoms [3]. Although the worldwide incidence of malaria is declining, there are still more than 200 million new cases and over half a million deaths from malaria yearly of which 90% were due to *P. falciparum* [1]. The decline in mosquito vector density and the scale-up of malaria control interventions contribute to the more than 25% decline of malaria transmission globally and even more in sub-Saharan Africa. The drop in the incidence of malaria increases the risk of misdiagnosing febrile illness thus resulting in a paradoxical increase in the demand for accurate diagnosis [4]. Over-diagnosis of malaria is common especially in patients from areas with low to moderate transmission rates. The consequence is failure to adequately treat other causes of fever [5].

The two diagnostic methods for routine detection of malaria parasites are microscopy and rapid diagnostic tests (RDTs). The choice between using microscopy or RDTs is dependent on several factors including local malaria epidemiology, skills of interpreters, caseload, and availability of microscopy for differential diagnostics. High-quality microscopy still remains the gold standard providing parasite quantification, allowing species and stage differentiation, enabling monitoring of antimalarial drug response and identification of other disease causing agents. Furthermore, in areas where the caseload of febrile patients is high, microscopy is a more cost-effective diagnostic option compared to RDTs [6], [7]. Conversely, the sustainability of microscopy requires skilled microscopists, maintenance of infrastructures, capital investment as well as quality assurance and control. In addition, malaria microscopy is time-consuming and labor-intensive and associated with inter- and intra-observer variability [8].

According to recommendations, at the community level or outside formal health care services where high quality microscopy generally is not available, RDTs may be feasible for diagnosing malaria [6]. Advantages of RDTs include the technical easiness of the tests, limited training requirements, simplicity of interpretation and absence of need for electricity. The sensitivities and specificities of RDTs are variable and heat instability as well as limited shelf life of the tests is a concern [3].

Microscopy can be cost-effective and multipurpose. Although quality assurance programs have now been established; the quality of microscopy-based diagnosis is frequently inadequate [7], [9]. At the primary health care level the excessive workloads of microscopists are a major contributor to poor performance and the sensitivity decreases especially when large numbers of samples are processed [8]. Typically, if performed according to recommendations, it takes 30 seconds to read a strongly positive malaria slide and 6–20 minutes to read a weakly positive or a negative slide [10]. In addition, average time to count parasitemia is 15 and 17 minutes for an expert and qualified microscopist respectively if performed according to recommendations [11]. Within external quality assessment programs in public health laboratories in Africa, the accuracy of microscopy performed by human observers were low and only 51% of parasite quantification results were considered acceptable [12].

Recent development in imaging techniques enables digitization of large parts of a microscope sample or even an entire blood film at a high optical resolution, e.g. 20×, 40×, or even >63× with oil immersion. The obtained digital sample can then be viewed on a computer screen in a manner similar to conventional microscopy, i.e. panned and zoomed to different magnification [13]. Computer vision-based detection and quantification of malaria parasites in digital images of blood films has been attempted in several previous studies using image features such as color, texture and shape in combination with machine learning [14], [15]. From a computer vision perspective the detection of malaria parasites in blood films is a complex task and parameters that need to be accounted for include e.g. blood film staining quality, illumination differences, image acquisition, image pre-processing, feature selection and classification [14].

Most of the published computer vision algorithms for identification of malaria parasites have been developed and tested using a relatively small number of patients and, due to different study end-points, comparisons between the performances of the algorithms are difficult to perform [14], [16], [17]. Furthermore, previous studies usually show results on an image level (i.e. microscope field-of-view) and are not attempting at patient level diagnosis [14].

We here propose and evaluate a novel diagnostic aid based on a computer vision algorithm that analyzes an average of more than 50,000 erythrocytes in a thin blood film, ranks sample areas according to probability of infection and presents a small subset (approx. 100) of the highest scoring areas as a single panel to the user. This means that the most suspicious regions from a sample area corresponding to 500–600 high-power fields-of-view in a microscope are collected into a panel corresponding to a single field-of-view. The tool can aid the human evaluator in the identification of *P. falciparum* ring-stage trophozoites and thus assist in the diagnostic process. Moreover, the method can also quantify the level of parasitemia and thus potentially provide a diagnostic system for antimalarial drug screening programs.

## Materials and Methods

### Giemsa-stained thin blood films

Routinely collected, anonymized thin blood films from 47 patients (27 cases and 20 controls) were obtained from the Helsinki University Central Hospital Laboratory (World Health Organization accredited laboratory for malaria diagnostics). Written informed consent was not required according to the Ministry of Social Affairs and Health, Finland's Act On the Medical Use of Human Organs, Tissues and Cells (Amendments up to 277/2013 included) because the samples collected contained no personal identity codes.

Thin blood films were prepared from EDTA-anticoagulated blood and Giemsa stained according to standard procedures [6]. All malaria samples included in the study had previously received a species-specific diagnosis of *P. falciparum*, as assessed with a conventional microscope by an expert microscopist according to WHO guidelines [6].

### Digitization of blood smears

**Figure 1:**
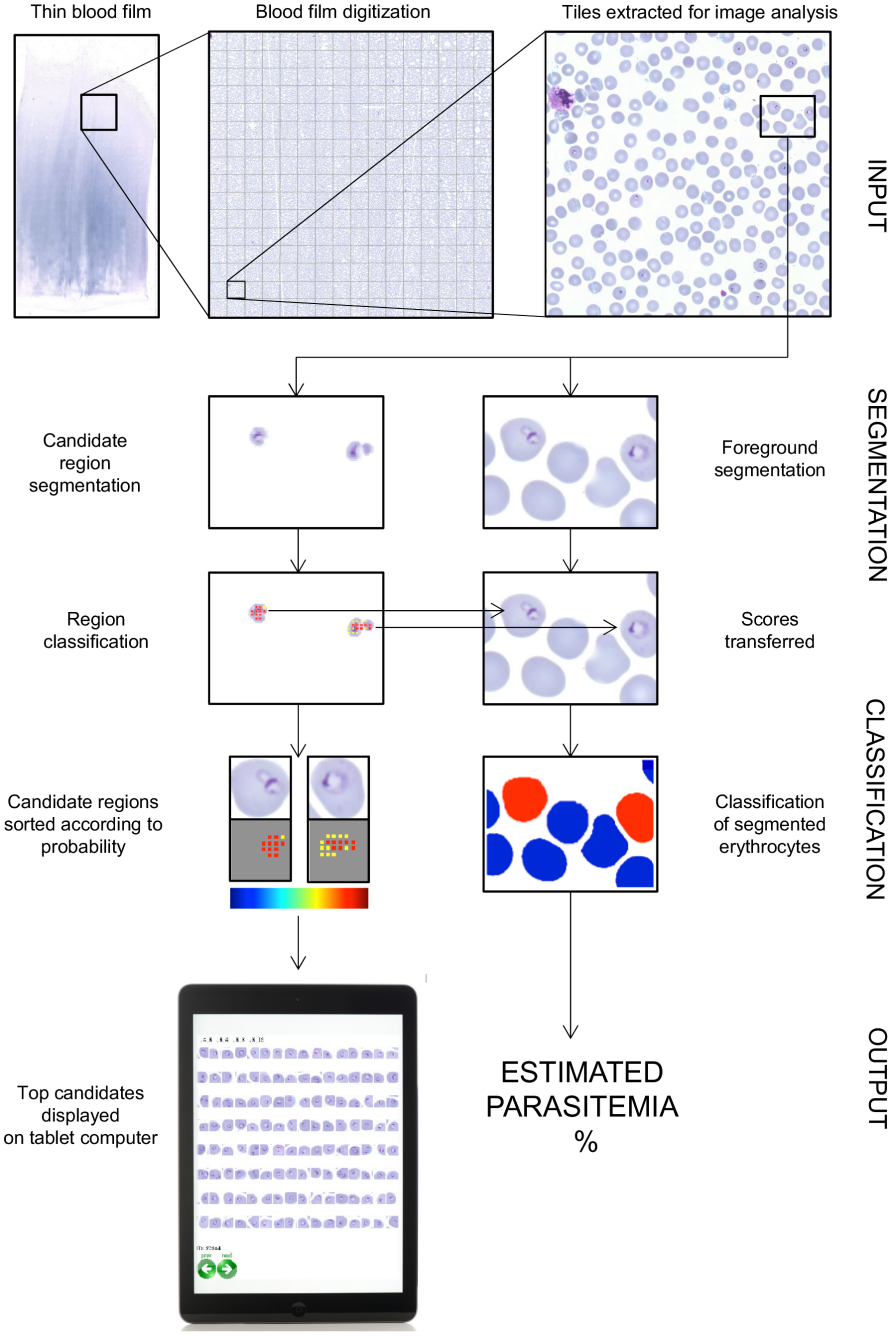
A flow chart of the proposed *P. falciparum* detection method resulting in a panel showing the most probable detections in one thin blood smear as well as the parasitemia count for the same sample.

According to recommendations 300–800 high power (100×, oil immersion) fields-of-view per thin blood smear sample should be screened to exclude a malaria infection [18], [19], which corresponds approximately to a sample area of approximately 6–20 mm^2^. For the current study, selected rectangular areas with an average size of 6 mm^2^ (range 5.28–7.40) from monolayer regions of Giemsa-stained thin blood films were digitized with a whole-slide scanning system that consists of a microscope (Axio Imager Z2, Carl Zeiss Microscopy AG, Jena, Germany) equipped with a motorized stage (SCAN 8, Märzhäuser, Wetzlar, Germany), a 63× oil immersion objective (Plan-Apochromat; numerical aperture 1.4, Zeiss Microscopy AG, Jena, Germany), an 1.0 camera adapter, an RGB LED illuminator (Tofra RGB LED, Tofra Inc, Palo Alto, California, USA), a microscope camera (CoolCube 1 with a 2/3″ charge-coupled device sensor with a pixel size of 6.45 µm, MetaSystems, Altlussheim, Germany) and software for whole slide scanning (Metafer 4, MetaSystems, Altlussheim, Germany). The scanner captured 475–667 (average 549) fields-of-view (pixel dimension 1280×1024; pixel size 0.10 micrometer) per sample and these were stored as uncompressed R, G and B channel TIFF (Tagged Image File Format) image files and then stitched and compressed into an ECW (Enhanced Compressed Wavelet, Intergraph, Intergraph, Norcross, GA) file format and uploaded to a whole-slide image management platform (WebMicroscope, Fimmic Oy, Helsinki, Finland) running image server software (Erdas Apollo Image Web Server, Intergraph, Norcross, GA). Finally the images were annotated and for the analysis downloaded as 1500×1500 pixel tiles from the server in a lossless Portable Network Graphic (PNG) format (Fig. 1).

### Image data sets and annotation of malaria parasites

The digitized blood films (n = 47) were divided into three separate sets; a training set (n = 10), validation set (n = 6) and test set (n = 31). The training set consisted of five thin blood films with a malaria infection and five uninfected control samples. The validation set consisted of three infected and three uninfected blood films and was used for optimization of the algorithm. Finally, the test set consisted of blood films from 19 patients with a malaria infection and 12 uninfected controls and used for assessment of accuracy.

Erythrocytes suspected to be infected were manually labeled by one of the researchers (N.L.) into two classes: certain parasites (label 1) or uncertain parasites (label 2) using a previously described annotation tool [20]. The number of annotated, certain parasites was 8329, 569, and 8093 in the training, validation and test set respectively. Only certain parasites (label 1) were considered when calculating the ground truth parasitemia. The digitized areas of the thin blood films are stored in a database and available for viewing at http://fimm.webmicroscope.net/research/momic/mamic

### Malaria parasite detection

The parasite detection method consists of two stages: 1) a candidate region segmentation and 2) *P. falciparum* detection. The rationale of the selected two-step approach is to first identify potential parasites using simple morphological parameters such as color, shape and size. Further detailed inspection of the remaining candidate regions is then performed with computationally more expensive algorithms aiming to detect the actual malaria parasites among the candidates (Fig. 1).

#### Candidate region segmentation

**Figure 2:**
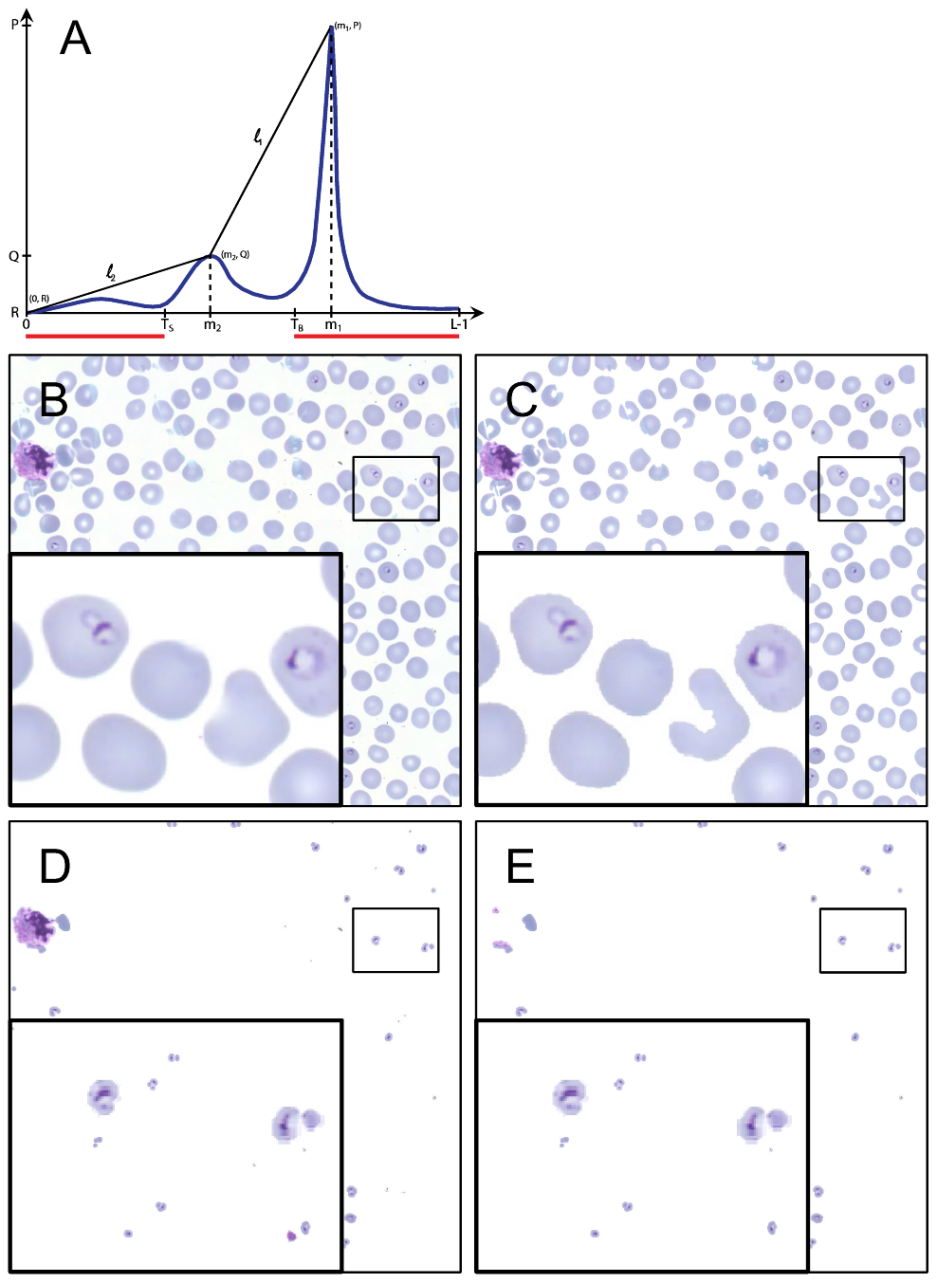
The candidate region segmentation phases: A) a typical bimodal histogram and definition of thresholds T_S_ and T_B_, B) an example tile segmented into C) foreground and background, D) strongly stained regions based on the histogram and E) the remaining candidate region after filtering out the smallest and the largest objects.

The green channel from each of the 1500×1500 pixel PNG tiles captured at 63× magnification of the Giemsa stained blood films was first smoothed using a median filter (3×3). The histograms *h* of has a bimodal distribution where the first mode m_1_ represents the background with maximum value *P*, while the second mode m_2_ represents the presence of red blood cells with maximum value *Q* (Fig. 2). Two thresholds were defined using auxiliary lines *l*_1_ and *l*_2_. The value that maximizes the distance between *l*_1_ and *h* was defined as *T_S_*, similarly the value that maximizes the distance between *l*_2_ and *h* was defined as *T_B_* as previously described [21].

*T_B_* separates an image into background (≥T_B_) and foreground (<T_B_) regions. While *T_S_* defines strongly stained regions (<T_S_) in the foreground.

From the foreground (<T_B_), all objects with an average area less than 10 µm^2^ e.g. platelets and debris were removed. Further, from the strongly stained regions of the foreground (<T_S_), objects larger than (40 µm^2^) were filtered out, e.g. leukocytes, aggregated platelets. The remaining strongly stained regions in the foreground defined the parasite candidate regions, which typically represent only a small fraction of the whole 1500×1500 pixel tile (for example 0.64% of the tile in Fig. 2).

#### Sliding window classification

**Figure 3:**
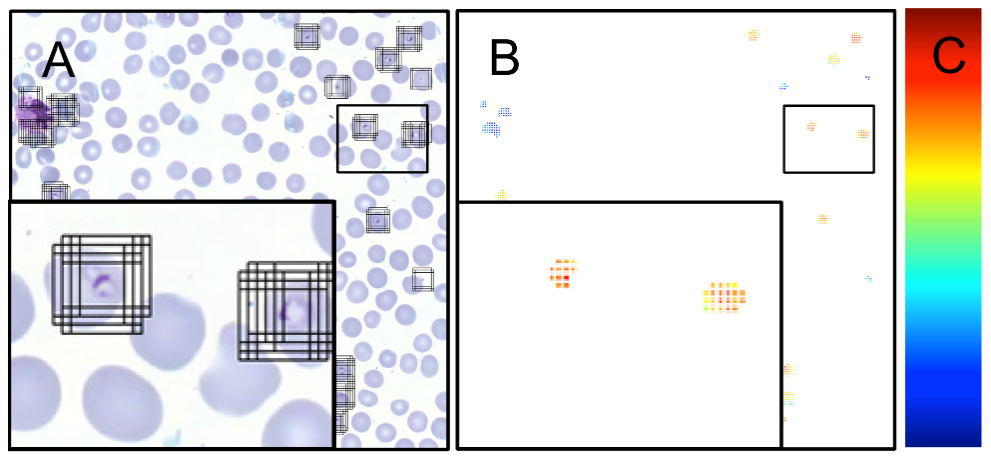
The sliding window support vector machine classification illustrated by A) an example tile showing all the windows extracted from the tile based on the candidate region segmentation, B) the resulting heat map after classification, in which the decision scores for the windows are visualized as small squares colored according to a color map and C) a bar showing the heat map for the classifier score values.

Each candidate region was divided into partly overlapping image windows of 64×64 pixels from which the below described features were extracted and used as input to the support vector machine (SVM) classifier. The windows were defined by their center points, which were sampled from the candidate regions using a sliding window-sampling step of eight pixels (Fig. 3). The size of the window was set to fit the largest parasites and also to capture their context. The size of the window was set to 6.4 µm×6.4 µm (64×64 pixels).

#### Features

The characteristics of the windows were mathematically modeled using a joint distribution of local binary pattern and rotation invariant local contrast features (LBP/VAR) and scale invariant feature transform (SIFT) features. Both LBP/VAR and SIFT features are widely used for object and face recognition in state-of-the-art computer vision applications [22], [23]. We hypothesized based on previous experience [24] and the literature that the combination of these features would be an accurate descriptor for detecting the parasites; LBP/VAR capturing the micropatterns and SIFT capturing the local gradients of the objects respectively.

The LBP operator compares each image pixel to *P* pixels in a neighborhood with a radius *R*. The intensity value of the central pixel is used to threshold the neighborhood pixels, thus forming a binary code. The LBP operator detects variations in the structure of the texture pattern, e.g. line ends, dots, borders, and curves. LBP is combined with a rotation invariant local contrast (VAR). The VAR represents the variance of the gray values of the neighborhood pixels. The LBP codes and the quantized VAR values were combined to an LBP/VAR feature vector. In the current study, the LBP and VAR features were computed from the gray scale images using parameter values P = 16 and R = 2 and with mapping to uniform and rotation invariant LBP patterns and VAR values quantized to four levels [25]. These parameters resulted in a LBP/VAR vector of 72 bins that was normalized to unit length.

The SIFT descriptor is an image region descriptor capturing the local image gradients. The descriptor is a three-dimensional histogram composed of gradients in quantized orientation. For the purpose of the current study, he SIFT descriptors were computed using the settings originally proposed [26], i.e. each window was divided to 4×4 subregions with eight orientation planes, resulting in a feature vector with 128 bins. The SIFT descriptor was computed from gray scale, saturation and hue channels and the final SIFT vector therefore consisted of 385 bins (128+128+128). An open source computer vision library was used to compute the features [27].

#### Classifier

A linear support vector machine (SVM) classifier [28] and a homogeneous kernel mapping [27] was used to classify the windows for the presence of malaria parasites based on the LBP/VAR and the SIFT features. The kernel mapping is a linear approximation of homogeneous kernels, which are commonly used in computer vision applications. Mapping of the χ^2^ kernel was applied to the features. Before the feature mapping the final feature vectors had 456 bins (72+128+128+128), and 3,192 bins after the mapping.

### Training of the classifier

The training set comprised of five patients with different parasitemia levels (7–16%) and five uninfected cases. To ensure a sufficient number of parasite examples, cases with high parasitemia counts were selected for training. The training samples correspond to windows (64×64 pixels) representing parasites or non-parasite objects (e.g. debris, platelets etc.) present in the candidate region.

The positive training samples, i.e. windows containing a parasite, were extracted from the malaria-infected cases using an annotation mask (analogous to the candidate region mask). The annotation mask was defined by expanding the annotation points that were assigned by the human observer approximately at the center of the trophozoite to circles of 16 pixels in diameter. In addition, the positive samples were extracted in four different orientations: (0°, 90°, 180°, 270°). Only annotations labeled as a certain parasite (label 1) were used for training and annotations labeled as an uncertain parasite (label 2) were excluded from the training set. Twenty thousand malaria positive training windows were chosen randomly within each of the positive training samples (n = 5) and, resulting in a total of 100,000 windows containing a parasite.

Ten thousand malaria negative training windows were extracted from the candidate regions of all ten training samples (randomly from both the infected and uninfected patient samples), giving a total of 100,000 malaria negative windows. Areas near an annotation labeled as a parasite were excluded when extracting negative training samples, so that positive and negative training windows could maximally have an overlap of 5%.

The total number of training windows from which the LBP/VAR and SIFT features were extracted was therefore 200,000, with 50% positive and 50% negative windows.

The cost parameter C of the linear SVM classifier was optimized on windows extracted from the three infected and three negative controls in the validation set with exponentially growing parameter values: C = (2^−10^, 2^−8^, …,2^8^,2^10^). The parameter was evaluated based on areas under the receiver operating characteristics (ROC) and precision recall (PR) curves for classification on a window level. The best performance on the validation set was achieved with a C value of 2^4^.

### Detection and visualization of classification scores

The sliding window classification was used to process the candidate regions in the test set, resulting in a classification score for each window, which form a dense classification map (Fig. 3). The classification map was visualized with a heat map, i.e. color-coded representation of classification scores, where a classification score of a window is shown as a colored square located in the center point of the window (Fig. 3).

A parasite induces multiple strong classification scores in overlapping windows near the parasite. To avoid multiple detections, non-maximum suppression (NMS)was applied to the classification maps. Thus, the highest classification scores were identified and scores closer than 4 µm (40 pixels) to the local maximums were suppressed. The remaining windows with associated classifications scores were entitled detections. The detections were ranked from the highest to the lowest according to the classification score and thereby the probability of containing a parasite.

### Evaluation of the tool on a patient level

**Figure 4:**
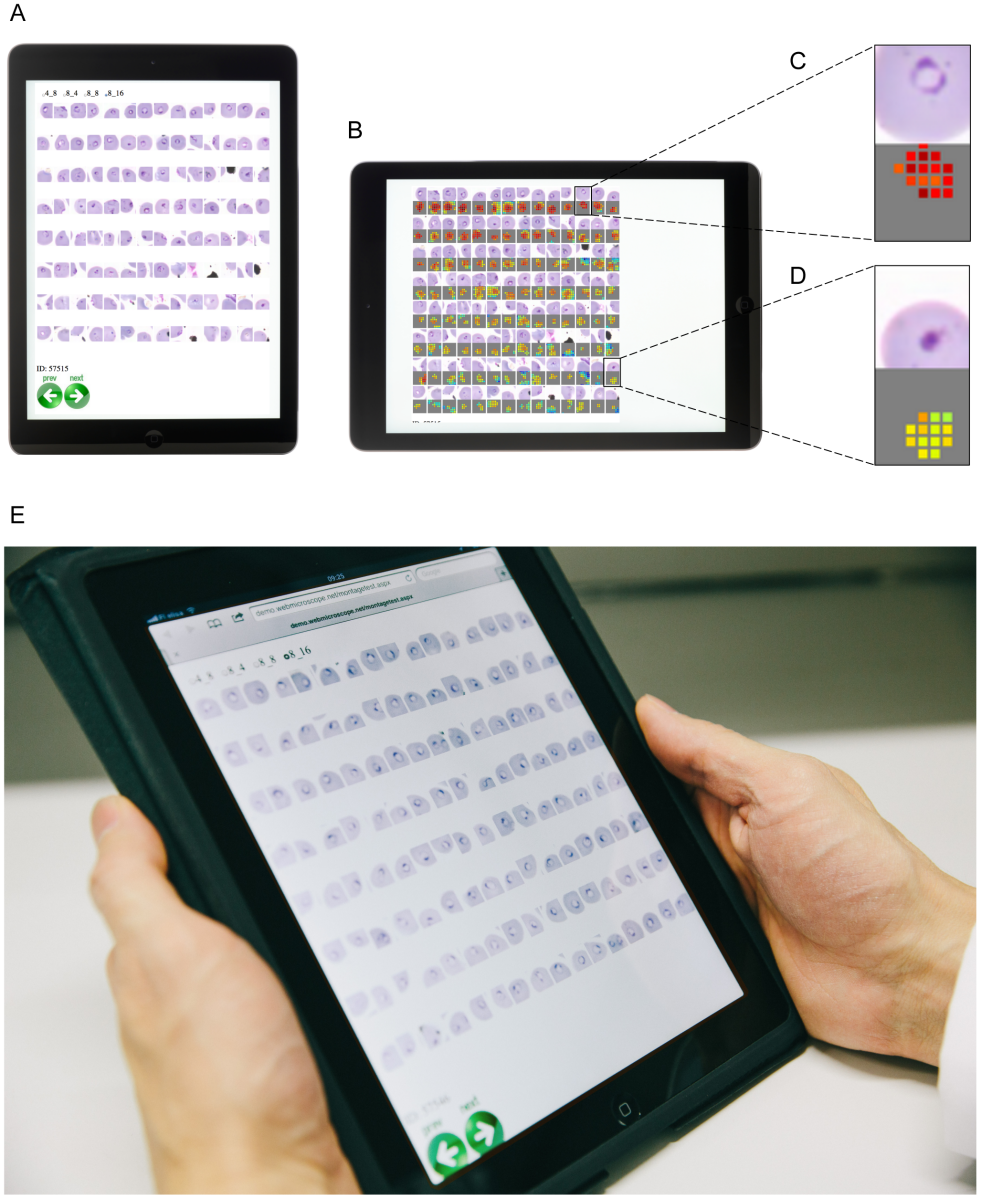
The reader assessment mode displayed on a tablet computer. A) A portrait view showing the highest ranked *P. falciparum* detections, B) the highest ranked detections and their corresponding heat maps viewed in a landscape mode, C) an example of a strong detection highly suspicious for *P. falciparum* and D) a lower ranked detection not suspicious for *P. falciparum*, E) a panel of the highest ranked areas displayed on a tablet computer.

To interpret the decision support tool on a patient level, two expert malaria microscopists evaluated the 128 highest-ranking detections in the test set samples (19 malaria infected cases and 12 uninfected controls) on a tablet computer (iPad, Apple Inc., Cupertino, CA, USA) (Fig. 4). The detections were arranged into 8×16 panels (http://demo.webmicroscope.net/montagetest.aspx) and presented to the expert in a random order blinded from the ground truth. No time limit for evaluation was set but the experts received a few of minutes of introduction to the decision support system before starting the interpretation. The introduction to the expert microscopist included guidance regarding how to swipe from one case to the next on the tablet computer and advice for viewing the detection areas and corresponding heat maps.

### Estimation of parasitemia

Accurate segmentation of erythrocytes is necessary to assess parasitemia i.e. the ratio of infected erythrocytes. Automated erythrocyte segmentation was applied to the malaria-infected samples to define a subset of well-defined erythrocytes. We decided to segment only well-defined erythrocytes since segmentation of clustered cells requires computationally expensive algorithms and approximations that introduce uncertainty to the infection status of individual cells. The erythrocytes were segmented from the earlier defined foreground mask (Fig. 2) as previously described [21]. First, in the strongly stained foreground (<T_S_), large objects (larger than 40 µm^2^) were removed. Second, objects with a roundness, 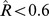 detected in the entire (<T_B_) foreground were removed from the mask, after which only the objects with a defined roundness and size remained. This was done to ensure that the detected cells represent only individual erythrocytes. Roundness 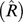 of an object was defined as a function of the area of the object (A) and perimeter (*P*): 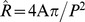. Third, objects with an area that deviated more than a standard deviation *σ* (0.06 micrometer) of the average erythrocyte size (7.52 micrometer) in the training set were removed.

**Figure 5:**
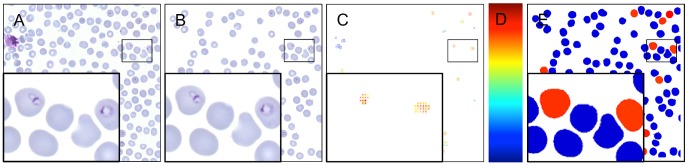
Parasitemia is estimated by first segmenting a set of erythrocytes and then scoring them based on the heat map e.g. the sliding window classification results. A) An example tile, B) segmented erythrocytes, C) the corresponding heat map, D) a bar showing the heat map for the classifier score values and E) the resulting erythrocyte classification to either *P. falciparum* infected (red) and uninfected erythrocytes (blue).

After the segmentation, the erythrocytes were scored based on the classification scores that were previously calculated for the detection of parasites (Fig. 5). The classification scores within a segmented erythrocyte were sorted and an average of the eight strongest scores was calculated. If there were less than eight classification scores in an erythrocyte, the average was calculated from the available ones. Erythrocytes that did not contain any classification score were assigned a score -∞. This value defined an erythrocyte score, which was used to classify the cells either as malaria infected or uninfected. The threshold for the erythrocyte score was obtained with a cross validation on the training samples: A threshold was set based on five random samples and then evaluated on the remaining samples in the training set. One thousand random sets were separately selected and optimized according to the F-measure.

### Statistical analysis

The detection accuracy of the classifier was evaluated with the area under the ROC curve (*AUC_ROC_*) and area under PR curve (*AUC_RR_*) respectively. The ROC curve is defined with false positive rate (FPR, 1-specificity) on the x-axis and true positive rate (TPR, sensitivity or recall) on the y-axis, and the PR curve is defined as a curve with TPR on the x-axis and positive predictive value (PPV or precision) on the y-axis. The F-measure is defined as a harmonic mean of precision and recall: 2*(precision*recall)/(precision+recall) and used in selection of the threshold for erythrocyte classification. The above-mentioned metrics are based on the outcomes of a binary test (true positive (TP), false positive (FN), true negative (TN) and false negative (FN)): FPR = FP/(FP+TN), TPR = TP/(TP+FN) and PPV = TP/(TP+FP). Respectively, the accuracy is calculated as a ratio: (TP+TN)/(TP+FP+TN+FN) and negative predictive value (NPV) as a ratio: TN/(TN+FN). The agreement between the diagnostic tool and automated methods in the assessment of erythrocyte and patient-level infection status was estimated by percent-agreement and kappa-statistics. Kappa values were categorized as suggested previously in the literature: <0 as disagreement, 0–0.20 as slight, 0.21–0.40 as fair, 0.41–0.60 as moderate, 0.61–0.80 as substantial, and 0.81–1 as almost perfect agreement [29]. Correlation coefficients were calculated using the Pearson product-moment correlation method.

### Ethics statement

This manuscript reports an explanatory retrospective analysis of routinely collected blood slides for malaria diagnostics at a reference laboratory (The Central Laboratory for the Hospital District of Helsinki and Uusimaa, HUSLAB, Helsinki, Finland). The Central Laboratory for the Hospital District of Helsinki and Uusimaa, HUSLAB, Helsinki, Finland approved the study protocol (VLE82M0005). According to the Ministry of Social Affairs and Health, Finland Act On the Medical Use of Human Organs, Tissues and Cells (Amendments up to 277/2013 included), written informed consent was not required because no clinical records were retrieved and the study contained no personal identifiers.

## Results

The proposed method is evaluated from two perspectives: 1) as a decision support tool that detects parasite-like objects in a digitized thin blood film and presents the most suspicious sample regions to a human observer and 2) as an automated parasitemia estimation tool by classifying segmented erythrocytes as infected or un-infected.

### The decision support system for malaria diagnosis

**Figure 6:**
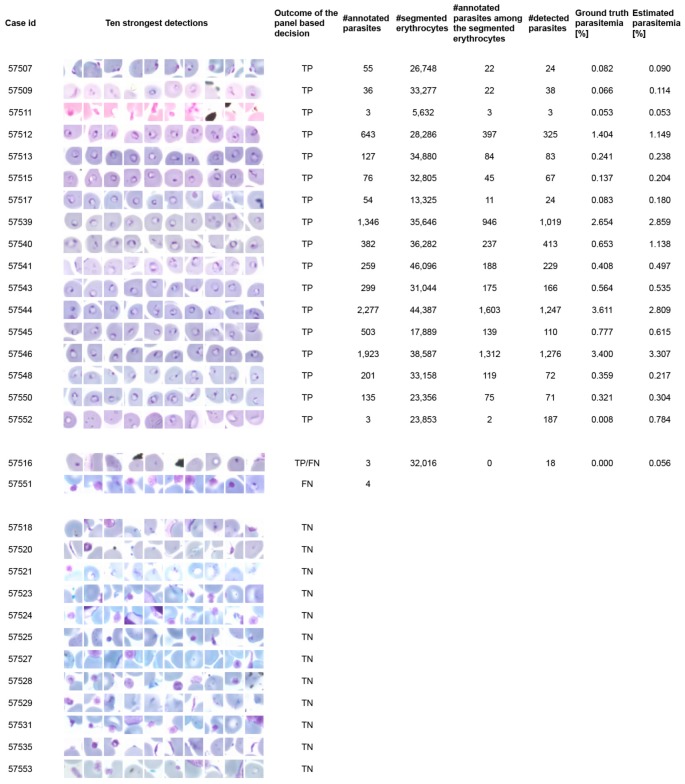
Ground truth annotations and outcome of the computer vision-assisted decision support method in each digitized thin blood film in the test series (19 malaria infected samples and 12 uninfected controls). Thumbnail pictures show for each patient ten of the 128 sample areas (i.e. detections) with highest probability of malaria infection detected by the image analysis algorithm and presented to the expert in the panel view described in Figure 4. Parasitemia was only calculated for cases considered as malaria positive based on visual inspection of the highest scoring detections. Note that only part of the erythrocytes were successfully segmented by the algorithm and therefore also the ground truth annotations in the segmented cells is lower than the total number of annotated parasites in a sample.

To evaluate the malaria algorithm on a patient level the panels with the 128 highest detections out of an average of 5,782 detections (range 1,793–12,975) in the 31 samples (19 malaria positive cases and 12 uninfected controls) were shown separately to two skilled malaria microscopists on a tablet computer (Fig. 4). The microscopists were then asked to make a decision on a sample level, if the patient had a malaria infection or not. The diagnostic sensitivity of the human observers on a patient level using the diagnostic tool was 95%; CI95% (81–95) (one false negative) and 90%; CI95% (75–90) (two false negatives) and the specificity was 100% for both readers. The accuracy of the decision support system was 97%; CI95% (80–97) and 94%; CI95% (76–94) for the two readers respectively and the agreement between the microscopists 97%; CI95% (81–100), (*κ* = 0.94). The ten strongest detections in each of the samples are shown in Fig. 6 and the full panels can be viewed at http://demo.webmicroscope.net/montagetest.aspx.

### Automated parasitemia estimation

For parasitemia estimation, automated erythrocyte segmentation was applied to the malaria-infected samples and the segmented cell was classified into infected and not infected. On average 29,066 (range 5,632–46,096) erythrocytes per sample were segmented, representing approximately 56% of all erythrocytes in the samples.

The sensitivity of the erythrocyte level classification was 84.9% (95% CI 78.5–91.3), the specificity 99.9% (95% CI 99.8–100.0) the negative predictive value (NPV) 99.9% (95% CI 99.8–100.0) and the positive predictive value (PPV) 74.2% (95% CI 61.5–86.9) respectively.

**Figure 7:**
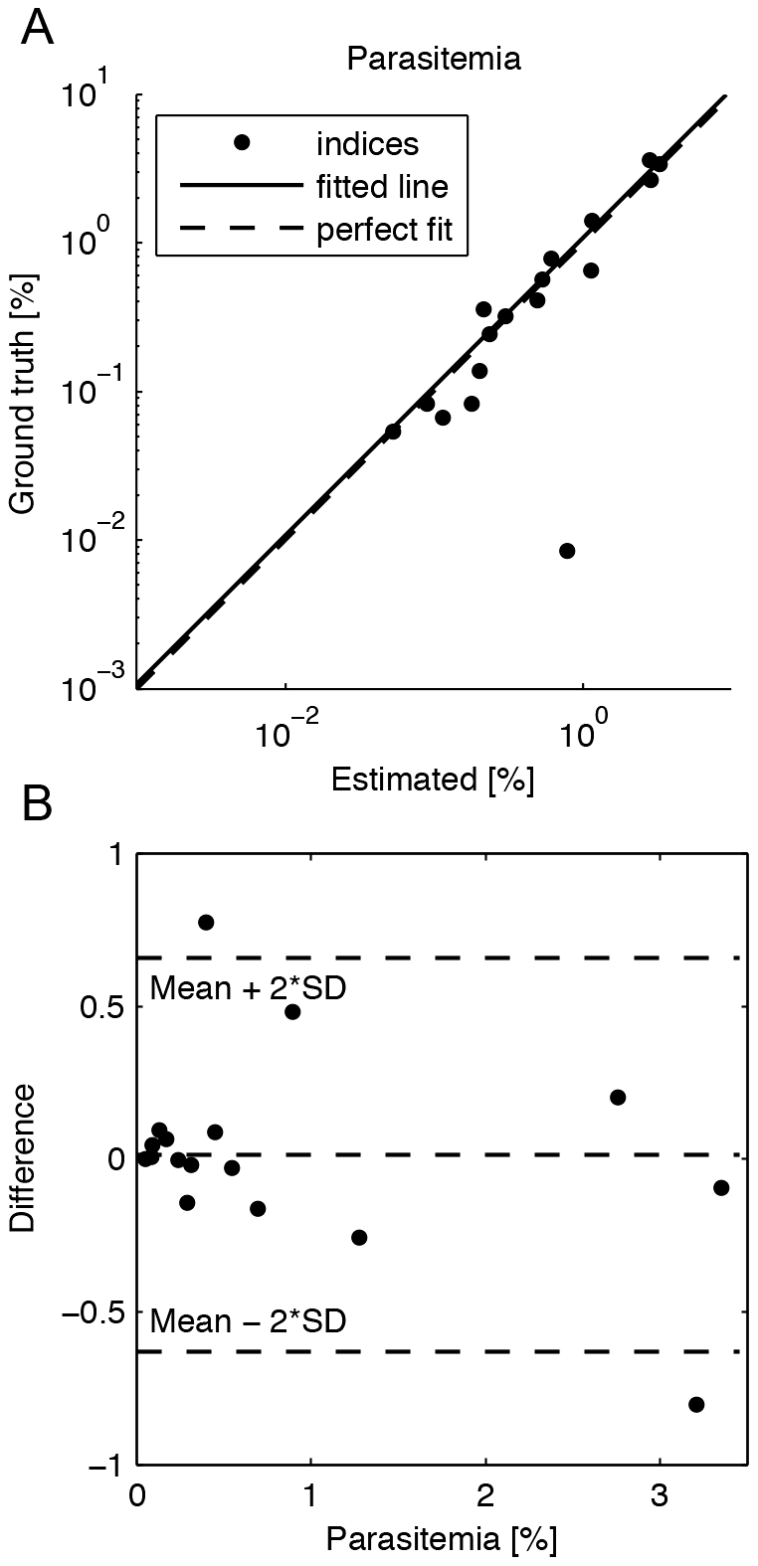
The agreement between the estimated parasitemia and ground truth is shown with A) a logarithmic agreement plot and B) a Bland-Altman plot.

The corresponding agreement between the human observer and the algorithm on a sample level as measured by the kappa test was 0.84. The AUC_ROC_ and AUC_PR_ of the classification was 0.997 and 0.901 respectively. Based on the classification results, parasitemia was calculated for all test samples as a ratio of infected erythrocytes to all segmented erythrocytes and the correlation coefficient between the automated and human observer-based parasitemia counts was 0.97 (Fig. 7). Only certain parasites (label 1) were considered when calculating the ground truth parasitemia.

Two clear outliers are seen in the parasitemia calculation (Figure 6 and 7). The first sample (no 57552) is an outlier in both of the plots (in 7A and in the top left corner of 7B), whereas the latter sample (no 57544) is the bottom right outlier in 7B. In the first sample (parasitemia overestimation) there is a lot of fine debris on top of the erythrocytes and in second sample (parasitemia underestimation) there are a substantial number of weakly stained, thin throphozoite rings that the algorithm misses (for sample details please see: http://fimm.webmicroscope.net/research/momic/mamic).

## Discussion

The present study describes a computer vision assisted method for screening of digitized thin blood films to detect ring-stage malaria parasites (early stage trophozoites of *P. falciparum*). A decision support system, which aids the microscopist in malaria diagnostics, is proposed. We show that image analysis combined with visualization of selected sample areas and a human expert decision on diagnosis is feasible and in the current patient series achieves an accuracy comparable to the malaria diagnostic quality criteria defined for a qualified microscopist [12]. In our approach, we do not aim to make the final diagnosis by a fully automated method, but rather present regions of interest in the digitized thin blood film that are indicative of a malaria infection and thus support the microscopist in performing the diagnosis.

In our study, each of the two experienced microscopists evaluated a panel of the 128 top ranking detections out of an average of approximately 6.000 detections identified by the algorithm in each thin blood film sample in the test set. The sample area presented in the panel corresponds approximately to one high power (100×) field-of-view in a conventional microscope (112×107 µm = 0.12 mm^2^) as compared to the total digitized area of a sample that contained in average 549 fields-of-view (6 mm^2^). The diagnostic tool achieved a sensitivity of 95% and 90% for each of the two microscopists, with a specificity of 100% on a patient level in the series of 19 malaria-infected samples and 12 uninfected controls.

Of the two microscopists who assigned the diagnosis using the decision support system, one made a single and the other two false negative decisions, i.e. they incorrectly interpreted positive malaria samples as uninfected. Both microscopists missed a malaria diagnosis in the same malaria positive case. The two misclassified samples are case numbers 57516 and 57551 and both had a very low number of annotated parasites, representing the second and third lowest levels of parasitemia in the test series (0.025–0.127%). (http://demo.webmicroscope.net/montagetest.aspx). The prior sample also contained debris and the latter a very high platelet count. One of the microscopists was doubtful regarding the diagnosis of this particular case and thus, in a true clinical setting confirmation would typically involve a tie-breaking smear read from the most senior microscopist, if the original two readers cannot agree on the sample positivity, species speciation, and parasitemia.

Most of the previous studies on computer vision applications for malaria diagnostics show results on an image (field-of-view) or erythrocyte level and only a few on a patient level. We were not able to find previous studies that would have used a semi-automated approach similar to ours, where a human observer makes the diagnostic decision based on screening with image analysis. In one study on a fully automated approach showing patient level results, thin and thick blood films from 174 patients were analyzed with a computer algorithm based on pattern, color and shape features, and a sensitivity of 92% and specificity of 90% was reported [30]. Direct comparisons with our results are difficult because both thin and thick smears, parasites in different stages and representing several different species were analyzed, whereas we studied ring-form trophozoites of *P. falciparum* in thin smears only. We decided to only analyze thin smears although we recognize that thick smears may have higher sensitivity based on human observer analysis. Thin blood films have a sensitivity limitation as compared to thick film reading, i.e. to obtain the same sensitivity as that for thick film at high power fields for ten minutes, a thin field must be examined for 30 minutes [18].

Another study on two sets of 20 and 41 thin smears from patients with early stage ring-form parasites reported a sensitivity of 100% in both series and specificities of 50% and 88%, respectively [31]. In a study on thin blood films from nine patients, also representing different species, the sensitivity of an algorithm based on area granulometry and a multi-class K nearest neighbor classifier was 72.4% and specificity 97.6% [32]. One could thereby argue that our semi-automated approach seems to be roughly on par with previously reported computer vision methods with regard to diagnostic accuracy on a patient level.

In contrast to the limited number of publications regarding the detection of malaria parasites on a patient level, there are a number of machine vision applications for detecting parasites on an erythrocyte level. Previous reports have included results on analysis of 2,000–30,000 erythrocytes for identification of malaria parasites [15], [31], [32] and some have used only some tens of microscope view fields for testing the performance [17], [32]. The sensitivity of the erythrocyte level detection of infection in our test set was 84.7% and the specificity 99.9%. A previous study, also in thin films, used a binary classifier to decide whether the erythrocyte is infected or not, followed by a multiclass classifier to assign the erythrocyte to a certain malaria infection life stage showed a sensitivity of 94% and a specificity of 99.7% based on 700 infected and 11.800 uninfected erythrocytes [16]. Our approach was to first segment strongly stained areas and then extract a series of features i.e. LBP/VAR and SIFT descriptors for classification. To our knowledge there is only one previous study in which texture features have been utilized for malaria detection [33]. In that study the algorithm was tested on a dataset that consisted of 825 erythrocytes representing different stages of both *P. falciparum* and *P. vivax* and achieved a sensitivity of 99.0% and a specificity of 99.8%. Our somewhat lower sensitivity as compared to other similar studies could partly be explained by the large number of erythrocytes analyzed - in average more than 50,000 erythrocytes per sample and an area that corresponds to 500–600 high-power (100×) fields of view in a conventional microscope, and a total of more than 900,000 cells.

In addition to aiding the malaria diagnosis on a patient level, the method described here allows automated calculation of parasitemia. Parasitemia was only calculated for cases that were considered positive based on visual inspection of the panel of sample areas with the highest probability of malaria infection. Our approach shows a high level of agreement (correlation coefficient 0.97) between the parasitemia as counted by a human observer and the level estimated by the computer vision method. Parasitemia was calculated on erythrocytes that were easily segmented, i.e. approximately 56% of erythrocytes. A potential drawback of our parasitemia estimation method is that if the segmented erythrocytes differ in their parasite count compared to the unsegmented erythrocytes, it might give rise to a biased parasite count. This is possible if parasite-containing erythrocytes are morphologically different compared to uninfected red blood cells. Other possible sources of bias in the parasitemia calculation include debris on top of the erythrocytes and throphozoite rings with aberrant morphology. It can be challenging for the algorithm to account for these artifacts and the problem might be best tackled by a general sample quality check, before running the parasitemia calculation.

Previous studies regarding automated calculation of malaria parasitemia reported correlation coefficients ranging from 0.97 to 0.99 [15], [16]. One study analyzed nine thin blood films including a total of 2,400 erythrocytes and compared the performance of a computer vision algorithm to human professionals. The algorithm, which included segmentation of nucleated components (e.g. white blood cells, trophozoites and gametocytes) and erythrocytes combined with object size based filters achieved a correlation of 0.97 with human observer based parasitemia assessement and a sensitivity of 97% [15].

Parasite clearance rates are essential for measuring treatment outcome of antimalarial drug efficacy particularly in clinical trials assessing artemisinin resistance. A robust and accurate automated method for determination of parasite clearance profiles could significantly reduce the workload also in this context, since readouts are usually performed by manual microscopy and requires at least four data points [34]. We therefore plan to evaluate the feasibility of the current method for parasite clearance assessment within a future field study.

The trophozoite ring-stage is the most common *P. falciparum* stage in a sample of peripheral blood from an individual with a malaria infection. The trophozoite ring-stage is the most common *P. falciparum* stage in peripheral blood samples from malaria infected individuals but late-stage tropohozoites, schizonts and gametocytes are occasionally seen. Thus, blood films with falciparum other than early ring stages as well a vast number of staining and sample qualities need to be trialed. Also, in future proof-of-concept studies, the time breakdown for capturing and processing the required number of images per slide, and how the method compares to routine slide examination should be addressed. Due to the visual similarity of the trophozoite in different malaria species, the method presented here most probably has the capability to also detect rings of vivax, ovale and malariae, but use of the method for speciation remains to be explored in further research.

Diagnostic tests for the detection of malaria should be rapid and have a high degree of accuracy. Furthermore, tests must be of low-cost, easy to use and easily available [9]. New molecular tests for malaria detection, such as RDTs and nucleic acid tests as well as mass spectrometry, have been developed. However, manual microscopy remains the gold standard diagnostic method for malaria [18]. An advantage of malaria microscopy is the multiplexed capacity to identify parasite co-infections such as trypanosomiasis and leishmaniasis. Other differential diagnosis such as lymphopenia or lymphocytosis, lymphoproliferative disorders, thrombocytopenia, and anemia (including sickle cell disease) can be made from a thin blood smear [35]. Multiplexed image analysis could allow several image analysis processes to be run in parallel with the ability to combine automated analysis of parasites with quantitative and morphological analysis of blood cells as an aid in the differential diagnosis of patients with fever. Automated microscopy of malaria parasites from digitized blood films has several advantages as compared to the approved manual approach. For example, the automated machine vision methods allow task shifting where a healthcare professional with limited expertise in microscopy can perform advanced analysis and receive instant diagnostic support [36].

We used a high-resolution scanning microscope with a 63× oil immersion objective to acquire the images. The digitization hardware is a challenge when implementing automated vision methods in low resource settings or in small and medium sized diagnostic centers in developed countries. The costs of high throughput scanners for sample digitization have so far prevented transfer of the new technology to low-resource settings. However, the system described here will be further assessed also in combination with less expensive digitization systems. Motorized scanning systems have been described that can be integrated with a conventional light microscope [37]Also, recent development allows decreasing the size of a microscope to construct miniaturized digital microscopy-imaging devices that are capable of producing high-resolution images [38], [39], [40], [41]. This can be achieved by the use of compact objective lenses combined with an image sensor [38] or with methods where the biological sample is placed close to the surface of an image sensor and illuminated with light emitting diodes [39]. Automated analysis can equally be applied to images generated by these devices.

An interesting alternative to our approach is a large-scale public experiment where images from malaria suspected blood films where classified by 1000 untrained, crowd sourced interpreters worldwide and combined into a diagnostic decision, resulting in an accuracy comparable to those of expert microscopists [42].

Positive effects of increased diagnostic throughput are improved case management, a more rational anti-malarial drug use, including increased access to treatments since a larger proportion of patients will be verified, and reductions in the development of resistance to antimalarial combination therapies. The massive investments in antimalarial drug development [43], need to be accompanied by a parallel commitment to improve diagnostic tools and their availability.

We present a novel system for decision support in *P. falciparum* microscopy using an automated machine vision method combined with a panel display showing regions of interest from a virtual thin blood smear of patients with suspected malaria infection. From each blood smear, an area equivalent to roughly 500 microscope high-power fields-of-view, covering an area with approximately 50.000 red blood cells, is compressed into a single easy-to-read panel showing malaria suspected areas of interest to aid the microscopist in assessing the malaria diagnosis.
